# Development and characterization of 96 microsatellite markers suitable for QTL mapping and accession control in an Arabidopsis core collection

**DOI:** 10.1186/1746-4811-10-2

**Published:** 2014-01-22

**Authors:** Patrick Cosson, Véronique Decroocq, Frédéric Revers

**Affiliations:** 1 INRA, UMR 1332 de Biologie du fruit et Pathologie, Villenave d’Ornon, F-33140, France; 2 Univ Bordeaux, UMR 1332 de Biologie du fruit et Pathologie, Villenave d’Ornon, F-33140, France

**Keywords:** Microsatellite, *Arabidopsis thaliana*, QTL mapping, Accession control

## Abstract

**Background:**

To identify plant genes involved in various key traits, QTL mapping is a powerful approach. This approach is based on the use of mapped molecular markers to identify genomic regions controlling quantitative traits followed by a fine mapping and eventually positional cloning of candidate genes. Mapping technologies using SNP markers are still rather expensive and not feasible in every laboratory. In contrast, microsatellite (also called SSR for Simple Sequence Repeat) markers are technologically less demanding and less costly for any laboratory interested in genetic mapping.

**Results:**

In this study, we present the development and the characterization of a panel of 96 highly polymorphic SSR markers along the *Arabidopsis thaliana* genome allowing QTL mapping among accessions of the Versailles 24 core collection that covers a high percentage of the *A. thaliana* genetic diversity. These markers can be used for any QTL mapping analysis involving any of these accessions. We optimized the use of these markers in order to reveal polymorphism using standard PCR conditions and agarose gel electrophoresis. In addition, we showed that the use of only three of these markers allows differentiating all 24 accessions which makes this set of markers a powerful tool to control accession identity or any cross between any of these accessions.

**Conclusion:**

The set of SSR markers developed in this study provides a simple and efficient tool for any laboratory focusing on QTL mapping in *A. thaliana* and a simple means to control seed stock or crosses between accessions.

## Introduction

Identifying plant genes that control key traits is central to understanding the mechanism of their gene action and to target them in breeding programs. Few genes controlling quantitative traits have been identified in plants and most have been cloned by linkage mapping [1]. Mapping of Quantitative Trait Loci (QTL) is a standard procedure that requires both genotypic (i.e. based on molecular markers) and phenotypic data generated from a segregating population [2,3]. In a first step, mapped QTL intervals of several tens of centimorgans are identified for which a portion of the phenotypic variation of trait is explained. A fine mapping approach that aims to narrow down these QTL to candidate genes is then implemented.

This initial step necessitates having a set of molecular markers distributed along the plant genome and polymorphic between the two parents of the progeny. In *Arabidopsis thaliana*, major inroads have been made for gene mapping, through the identification of thousands of Single Nucleotide Polymorphism (SNP) markers [4-11] or through extensive genome re-sequencing of individual accessions [12-14]. However, mapping technologies using SNPs markers are still rather expensive and not applicable in every laboratory. On the other hand, due to their relative abundance in the genome and simple cost effective detection microsatellite markers (also named simple sequence repeat or SSR markers) remain for many laboratories the markers of choice in a primary QTL mapping screen. Microsatellites are tandem repeat motifs of 1 to 6 bp that have frequent occurrence in all genomes analyzed to date [15,16]. The high variability of microsatellites is mainly due to different number of repeats in the region of the repeat motif but also to short insertion/deletion events [17]. Thus, SSR markers usually exhibit many alleles in comparison to SNP or other polymerase chain reaction (PCR)-based markers. Moreover, they have the advantage of being codominant. Consequently, only a limited number of markers are sufficient to detect polymorphism among parental lines. As the flanking regions of the repeat motif are in many cases highly conserved (particularly in the case of EST derived SSRs), microsatellite markers are easily and reproducibly amplified by PCR in most accessions and visualized by gel electrophoresis (agarose, polyacrylamide) or automated sequencing. In addition, their identification is relatively simple when plant genome sequences are available. In addition, in the case of *A. thaliana*, several studies showed that microsatellites are abundant and highly variable [18-20].

In this paper, we describe the development and the characterization of a panel of 96 SSR markers distributed along the *A. thaliana* genome suitable for QTL mapping in an Arabidopsis core collection.

## Results and discussion

### SSR development

**Figure 1:**
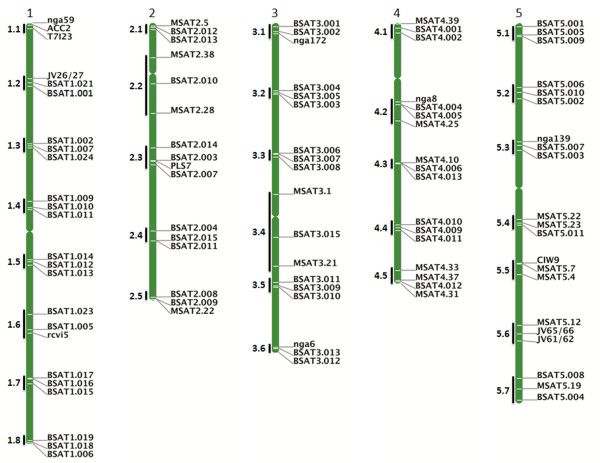
**Genomic localization of the SSR markers and the selected genome regions.** This map was made using the chromosome map tool from TAIR ( http://www.arabidopsis.org/index.jsp). Number at the top of each chromosome is the number of each Arabidopsis chromosome. Markers are indicated on the right of each chromosome. The genome regions selected in this study are indicated on the left of the chromosome in front of the corresponding markers.

**Figure 2:**
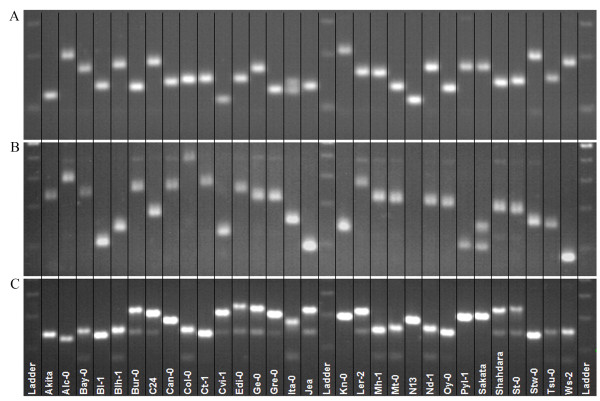
**Polymorphism obtained with three SSR markers visualized using agarose gel.** Agarose electrophoresis of the corresponding PCR products is shown for the thirty accessions (indicated at the bottom of the picture). **(A)** BSAT1.024; **(B)** MSAT3.1; **(C)** MSAT5.22.

The primary aim of this study was to produce molecular markers suitable for QTL mapping in *A. thaliana* without the use of expensive and specific technical platforms. Therefore, we decided to develop a panel of polymorphic SSR markers among accessions of the Versailles core collection 24 which maximizes genetic diversity in a limited number of individuals and has been used to generate F2 and Recombinant Inbred Lines (RIL) [21,22]. We showed previously in several studies that these accessions display a diversity in behavior toward plant viruses allowing development of mapping projects to identify plant genes involved in plant/virus interactions [23,24]. Our strategy was to identify markers distributed along the Arabidopsis genome, approximately every 4 Mb (approximately 20 cM), which are as polymorphic as possible among the Versailles core-24 accessions. Those criteria optimize their use for mapping analysis within progenies obtained from crosses between any of the 24 Versailles accessions [21]. Six other accessions (Col-0, C24, Ws-2, Nd-1, Bay-0 and Ler-2) were added to the study as they are frequently used as parents in our studies [23-27]. The criteria of 4-Mb intervals along each chromosome generated 8 regions (1.1 to 1.8) in chromosome 1 (chr1), 5 regions (2.1 to 2.5) in chr2, 6 regions (3.1 to 3.6) in chr3, 5 regions (4.1 to 4.5) in chr4 and 7 regions (5.1 to 5.7) in chr5 (Figure 1) in which we searched for tandem repeat motifs. Thirty-one SSR markers distributed throughout these regions were found in the Versailles MSAT database [28] or from The Arabidopsis Information Resource (TAIR) [29]. They displayed high genetic diversity (Additional file 1: Table S1) but they were not sufficient to cover the 31 genomic regions of interest (Figure 1). Moreover, they are not sufficiently polymorphic among our set of accessions (Additional file 1: Table S2). To complete this marker set, new loci containing tandem repeat motifs were identified within the Col-0 sequence as described in the Methods section. We preferentially selected perfect dinucleotide repeat motifs with a minimum of ten repeats except for a few regions where we had to select mono or trinucleotide repeat motifs or imperfect motifs (Additional file 1: Table S1). Primer sets were developed to amplify these repeat containing loci and tested among the 30 accessions. Our aim was to select SSRs in each region that displayed at least one polymorphism among all accessions and also for which polymorphism can be preferentially detected on agarose rather than polyacrylamide gels. Following this procedure, we generated 65 new SSRs, named BSATX.Y, X being the chromosome number and Y, the development rank number, with two to four markers generated in each selected chromosomal region. They are described in Additional file 1: Table S1 and their relative position on the five Arabidopsis chromosomes is presented in Figure 1. For some regions, especially those located around the centromere, we extended the size of the interval in order to identify suitable SSRs (Figure 1). Indeed, it has been previously shown that the occurrence of microsatellites is consistently lower in the centromeric regions than in the rest of the chromosomes [30]. To facilitate the use of these markers, primers were designed such that almost all SSRs can be amplified using the same PCR conditions. An example of SSR polymorphism displayed on an agarose gel is presented in Figure 2. The size of each SSR allele for each accession is presented in Additional file 1: Table S2. A few SSRs in some accessions could not be amplified at all, most likely due to polymorphisms or deletions in primer binding sites compared with the Col-0 sequence used for primer design. In other cases two amplified products were produced, most likely due to residual heterozygosity (Additional file 1: Table S2). From the size polymorphism of each SSR, several features were deduced. The average number of alleles for each marker is 14, ranging from 2 to 27 alleles per marker (Additional file 1: Table S1). The average polymorphism information content (PIC, which corresponds to the average allelic diversity of all the markers, see Methods) for the markers reaches 0.87 with values ranging from 0.50 to 0.96 (Additional file 1: Table S1) and the percentage of polymorphic markers between each pair of Arabidopsis accessions ranges from 64% to 99% with an average value of 90% (Additional file 1: Table S3). Thus, except for only a very few chromosomal regions, the SSR set distinguishes all the accessions (Additional file 1: Table S2 and Additional file 1: Table S4).

With this panel of SSR markers, any primary QTL mapping can be easily achieved with progenies (F2 or RIL populations) issued from any cross between the 24 Versailles accessions or between these accessions and Col-0, C24, Ws-2, Nd-1, Bay-0 and Ler-2. As soon as two accessions diverge for a trait of interest, the genetic factors associated with this phenotype can be located on the five Arabidopsis chromosomes. In addition, to simply revealing SSR polymorphisms, these SSRs are amplified using similar PCR conditions and subsequently displayed on agarose gels.

The high level of genetic diversity of these SSRs is in agreement with the high genetic diversity of the Versailles core collection [22,31]. Within the collection these 96 markers display more than 1,300 different alleles. This high level of polymorphism also makes them markers of choice for other Arabidopsis segregating populations which do not belong to the Versailles collection as illustrated in our recent genetic mapping study [24]. In addition, as only PCR and electrophoresis are needed to reveal polymorphism, this set of SSR markers has significant advantages and is complementary to the use of SNP markers [31,32] or Simple Sequence Length Polymorphism (SSLP) markers which are based on indels [33].

### Accession identification using a set of three highly polymorphic SSR markers

Due to the high level of genetic diversity displayed by the developed markers, we examined the possibility of differentiating each of the accessions with a minimum number of SSR markers. In this regard, we determined that using a select combination of only 3 SSR markers we could discriminate each accession from the 29 others. These markers are BSAT1.024, MSAT3.1 and MSAT 5.22 (Figure 2). Thus, these markers constitute a powerful tool to control seed stocks and crosses for the accessions studied in this work and likely for many other worldwide accessions as well.

## Methods

### Plant material

Accessions included in the Versailles core collection 24 were obtained from the INRA Versailles Centre [21]. Other accessions were obtained from the Nottingham Arabidopsis Stock Centre [34].

### Mining of microsatellites

The Arabidopsis BAC clones file containing the GenBank versions (fasta format) of sequences that make up the TAIR AGI map [35] was subsequently searched for the presence of one to three perfect repeat motifs using the Sputnik software [36]. The minimum length for each type of SSR is set as follows: mononucleotide repeats ≥30 nucleotides; dinucleotide repeats ≥30 nucleotides and trinucleotide repeats ≥24 nucleotides.

### Primer design and PCR conditions

Sequences of the SSRs that were identified in the genomic areas of interest were then subjected to similarity searches in the TAIR Whole Genome (BAC clones) dataset through the BLASTN algorithm. SSRs with several matches along the genome were eliminated. Oligonucleotide primers were designed for selected SSR loci using the PRIMER3 software [37] and were also subjected to BLASTN analysis. The parameters for primer design were as follows: amplicon size of 100–350 bp, primer size of 18–25 bp and primer melting temperature of 55–60°C with an optimum at 57°C.

Genomic DNA used for PCR was extracted from Arabidopsis young leaves using the NucleoSpin® Plant kit (Macherey-Nagel, Düren, Germany). PCRs were performed in a final volume of 15 μl consisting of 15 ng of DNA template, 1 U of Taq polymerase (Fermentas, Glen Burnie, MD, USA), 1x Taq reaction buffer, 0.2 mM dNTPs, 1.5 mM MgCl2, 200 nM of each primer. Conditions used for amplification were as follows: preincubation at 94°C for 2 min; followed by 35 cycles of denaturation at 94°C for 30 s; annealing at 57°C for the SSRs developed in this study, 50°C for the MSAT SSRs and 55°C for the other SSRs for 30 s; elongation at 72°C for 30s and a final extension step at 72°C for 10 min.

To reveal polymorphism, the PCR products were subsequently separated in 3% agarose gels. To determine the precise PCR product size, 6% acrylamide gels were used.

### Genetic diversity

Polymorphic SSR markers were scored for the presence or absence of the corresponding bands among the tested accessions. Stutter and background bands were excluded. The scores ‘1’ and ‘0’ indicated presence and absence of bands, respectively. The polymorphism information content (PIC) of each marker was calculated according to [38] as follows: PIC = 1-∑Pi^2^, where Pi is the band frequency of the i^th^ allele. PIC provides an estimate of the discriminatory power of a locus by taking into account, not only the number of alleles but also the relative frequencies of those alleles. PIC values vary from 0 (monomorphic) to 1 (very highly discriminative, with many alleles in equal frequencies).

## Supplementary Material

Additional file 1: Table S1 Description of the SSR markers. **Table S2:** Size of the SSR markers for all the 30 accessions. **Table S3:** Percentage of polymorphic markers between each pair of Arabidopsis accessions. **Table S4:** Non polymorphic regions identified among accessions. Click here for file
